# Postoperative Prognostic Nutritional Index and Fibrinogen Could Well Predict Poor Prognosis of Acute Type A Aortic Dissection Patients After Surgery

**DOI:** 10.21470/1678-9741-2022-0185

**Published:** 2024-02-06

**Authors:** Jia-Wen Hu, Tao Shi

**Affiliations:** 1 Department of Cardiovascular Surgery, First Affiliated Hospital of Medical School, Xi’an Jiaotong University, Xi’an, People’s Republic of China

**Keywords:** Airway Extubation, Prognosis, Fibrinogen, Afibrinogenemia, Odds Ratio

## Abstract

**Introduction:**

Inflammatory and immunological factors play pivotal roles in the prognosis of acute type A aortic dissection. We aimed to evaluate the prognostic values of immune-inflammatory parameters in acute type A aortic dissection patients after surgery.

**Methods:**

A total of 127 acute type A aortic dissection patients were included. Perioperative clinical data were collected through the hospital’s information system. The outcomes studied were delayed extubation, reintubation, and 30-day mortality. Multivariate logistic regression analysis and receiver operating characteristic analysis were used to screen the risk factors of poor prognosis.

**Results:**

Of all participants, 94 were male, and mean age was 51.95±11.89 years. The postoperative prognostic nutritional indexes were lower in delayed extubation patients, reintubation patients, and patients who died within 30 days. After multivariate regression analysis, the postoperative prognostic nutritional index was a protective parameter of poor prognosis. The odds ratios (95% confidence interval) of postoperative prognostic nutritional index were 0.898 (0.815, 0.989) for delayed extubation and 0.792 (0.696, 0.901) for 30-day mortality. Low postoperative fibrinogen could also well predict poor clinical outcomes. The odds ratios (95% confidence interval) of postoperative fibrinogen were 0.487 (0.291, 0.813) for delayed extubation, 0.292 (0.124, 0.687) for reintubation, and 0.249 (0.093, 0.669) for 30-day mortality.

**Conclusion:**

Postoperative prognostic nutritional index and postoperative fibrinogen could be two promising markers to identify poor prognosis of acute type A aortic dissection patients after surgery.

## INTRODUCTION

Acute type A aortic dissection (ATAAD) is a life-threatening cardiovascular emergency, which accounts for 58-62% of all aortic dissection (AD) with extremely high mortality and disability rates^[1]^. According to data from the International Registry of Acute Aortic Dissection, in-hospital surgical mortality rate could be as high as 30%, and the mortality rates after discharge range from 4-48% at the 1^st^ year and 9-63% at the 5^th^ year^[2]^. Therefore, it is important to accurately identify high-risk ATAAD patients by exploring the predictors of poor prognosis.

Accumulating evidence has confirmed that inflammatory and immunological factors are intimately involved in the progression and prognosis of ATAAD^[3,4]^. Inflammatory cell infiltration contributes to a sustained injury response, leading to medial degeneration and AD formation^[4]^. Several inflammatory factors, such as C-reactive protein, interleukin-6, tumor necrosis factor-α, and pentraxin-3, are increased in ATAAD patients^[5]^. The JAK2 gene, which is involved in the regulation of inflammatory response, was significantly downregulated in aortic specimens of ATAAD patients^[6]^. Anti-inflammatory liposome therapy alleviates aortic injury and prolongs survival time in both acute and chronic AD mice^[7]^. An Italian study found that T lymphocytes were reduced in the thoracic aortic specimens and peripheral blood of ATAAD patients^[5]^. Innate and cytotoxic cells are upregulated and are involved in the pathogenesis of ATAAD.

Due to this association, multiple systemic inflammatory and immune biomarkers have been studied in AD to predict its prognosis, including neutrophil-lymphocyte ratio (NLR), systemic immune-inflammation index (SII), and prognostic nutritional index (PNI). Higher NLR and SII were associated with adverse events in the hospital or during follow-up in AD patients^[8,9]^. Patients with a lower preoperative PNI showed significantly higher in-hospital mortality, a higher proportion of prolonged mechanical ventilation (MV), and longer intensive care unit (ICU) stay after surgery for ATAAD^[10,11]^. In addition, several new biomarkers derived from NLR were correlated with systemic inflammation and immune status and were good prognostic indicators of malignant tumors and cardiovascular diseases, including systemic inflammation response index (SIRI), advanced lung cancer inflammation index (ALI), and pan-immune-inflammation value (PIV)^[12,13]^. These indices outperformed other well-known peripheral blood parameters. However, it remains to be clarified whether these indices can act as prognostic biomarkers of ATAAD, and which one is optimal.

Therefore, the present study explored the predictive value of SIRI, SII, ALI, PNI, and PIV on delayed extubation, reintubation, and 30-day mortality. We further compared the sensitivity and specificity of these indices in the prediction of adverse outcomes. We aimed to identify the optimal indicator to guide risk stratification and treatment of ATAAD patients.

## METHODS

### Study Subjects

**Fig. 1:**
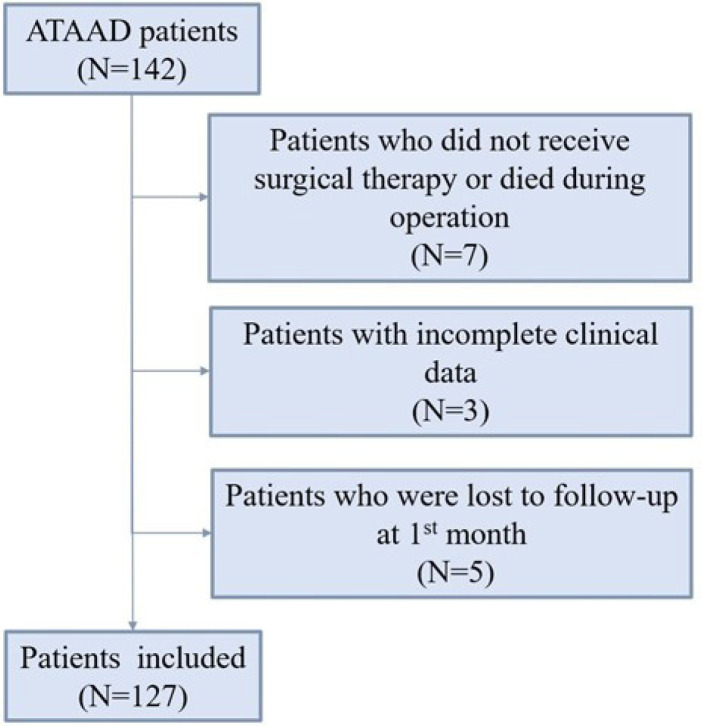
Flow chart of screening. ATAAD=acute type A aortic dissection.

Patients diagnosed with ATAAD from September 2020 to September 2021 were enrolled in this study. The diagnosis of ATAAD was confirmed by computed tomographic angiography. Patients who underwent no surgical treatment or who died during the operation were excluded. There were 142 ATAAD patients at first. Of these patients, seven were excluded because they did not receive surgical therapy due to aortic rupture or economic factors or died during the operation, three were excluded because some clinical data were missing, and another five patients who were lost to follow-up at the 1^st^ month after surgery were also excluded (Figure 1). The study was conducted in accordance with the Declaration of Helsinki and was approved by the Medical Science Research Ethics Committee of the First Affiliated Hospital of Xi’an Jiaotong University (No.2021-621), and individual consent for this retrospective analysis was waived.

### Data Collection and Definition

Perioperative clinical data of all patients, including demographic characteristics, laboratory parameters, surgical information, and detailed data of MV and reintubation, were retrospectively collected through the hospital’s information system. The prognostic indices included delayed extubation, reintubation, and 30-day mortality. Delayed extubation was defined as MV for > 48 hours. Patients were followed up at the 1^st^ month after surgery through re-examination in the outpatient clinic or telephone consultation. Body mass index (BMI) was calculated as weight/height2 (kg/m2). The immune-inflammation parameters were obtained according to the following formulas:

► NLR: peripheral blood levels of neutrophil count/lymphocyte count.
► SIRI: monocyte count × NLR.
► SII: platelet count × NLR.
► PNI: 10 × serum albumin (g/dL) + 0.005 × total lymphocyte count.
► ALI: BMI × blood albumin (g/dL)/NLR.
► PIV: platelet count × monocyte count × NLR.

### Surgical Technique

The operation was performed by a surgical team with the patient under general anesthesia. Cardiopulmonary bypass (CPB) was established at different sites according to the status of the patient (right axillary artery, femoral artery, innominate artery, and double arterial cannulation). Left radial artery and left dorsalis pedis artery catheterization for pressure measurement were performed. The patient was cooled to 28°C (nasopharyngeal temperature). The ascending aorta was clamped, and cold blood cardioplegia was infused through the coronary ostia to accomplish cardiac arrest. Antegrade cerebral perfusion for brain protection was established by axillary perfusion with a clamped brachiocephalic artery and direct cannulation of the left common carotid and subclavian arteries. The detailed operation procedure depended on the specific pathological changes of each patient, including Bentall procedure, David procedure, ascending aorta replacement + semiarch or total arch replacement, or Sun’s procedure (total arch replacement using a tetrafurcate graft with stented elephant trunk implantation). Some patients also concomitantly underwent coronary artery bypass grafting (CABG) and ascending-femoral bypass.

### Statistical Analysis

Statistical analyses were performed using IBM Corp. Released 2013, IBM SPSS Statistics for Windows, version 22.0, Armonk, NY: IBM Corp., MedCalc 18.2 (MedCalc statistical software, Inc., San Diego, California, United States of America), and GraphPad Prism 8.0 (GraphPad Software, Inc., San Diego, California, United States of America). Variable distribution was examined using the Kolmogorov-Smirnov test. Continuous variables are presented as means ± standard deviation for normal distributions and as medians (interquartile range) for skewed distributions. Percentages are given for categorical data. Differences of variables between groups were examined using Student’s *t*-test, Mann-Whitney U test, χ2 test, or Fisher’s exact test, as appropriate. Univariate and multivariate logistic regression analyses were used to screen the risk factors for poor prognosis. Receiver operating characteristic (ROC) analysis was used to assess the predictive performance of selected risk factors. Statistical significance was defined as *P*<0.05, and all results were two-tailed.

## RESULTS

### Baseline Characteristics of Participants by Clinical Outcomes

A total of 127 patients were included in this study. Ninety-four of them were male, and the mean age was 51.95±11.89 years. A total of 49.6% were hypertensive. The rates of delayed extubation, reintubation, and 30-day mortality were 43.7%, 16.8%, and 13.6%, respectively, in the present study. Eighty-six patients underwent ascending aorta replacement + Sun’s procedure, 24 underwent Bentall procedure + Sun’s procedure, six underwent David procedure + Sun’s procedure, five underwent Bentall procedure, three underwent Bentall procedure + semiarch replacement, two underwent ascending aorta + semiarch replacement, and one underwent ascending aorta replacement. In addition, seven patients underwent ascending-femoral bypass, and two underwent CABG. Eight patients who died or were discharged within 48 hours after surgery for personal reasons were excluded from the analysis of delayed extubation. Fourteen patients who had never been weaned from MV were excluded from the reintubation analysis.

The groups with different clinical outcomes (Table 1) had comparable baseline characteristics, except for a higher malperfusion rate in the delayed extubation group. Surgery time was longer in reintubated patients and patients who died within 30 days. The rate of ascending-femoral bypass was higher in patients who died within 30 days. Delayed extubation patients had a longer CPB time and a higher rate of David procedure. D-dimer and fibrinogen (FIB) degradation products at admission were significantly higher in patients who died within 30 days but lower in delayed extubation patients. We also found that postoperative FIB (postFIB) was significantly lower in delayed extubation patients, reintubation patients, and patients who died within 30 days (*P*-values 0.001, 0.001, and 0.003, respectively). Among all immune-inflammatory parameters (Table 2), preoperative SIRI and PIV were higher and PNI was lower in delayed extubation patients. The postoperative PNIs (postPNI) were significantly lower in longer MV patients, reintubation patients, and patients who died within 30 days (*P*-values 0.003, 0.027, and 0.009, respectively). Pre and postoperative ALI did not show significant differences between groups. These results indicated that postFIB and postPNI were intimately correlated with poor clinical outcomes.

**Table 1:** Baseline characteristics of acute type A aortic dissection patients by different clinical outcomes.

Index	Delayed extubation			Reintubation			30-day mortality		
	Yes	No	*P*-value	Yes	No	*P*-value	Yes	No	P-value
	(N=52)	(N=67)		(N=19)	(N=94)		(N=16)	(N=111)	
Age, years	51.71±11.24	51.54±11.43	0.934	54.53±11.79	51.18±11.43	0.249	52.31±11.41	51.39±11.54	0.765
Sex, male/female	36/16	53/14	0.219	12-jul.	73/21	0.182	11-mai.	83/28	0.607
BMI, kg/m^2^	26.29±3.91	25.32±3.73	0.176	25.75±3.84	25.80±4.14	0.962	27.23±5.21	25.84±3.89	0.233
HTN, %	48%	51%	0.773	53%	48%	0.705	56%	49%	0.570
DM, %	2%	3%	0.714	5%	2%	0.438	0%	3%	0.506
CKD, %	2%	7%	0.171	10%	4%	0.266	0%	6%	0.301
Smoking, %	35%	48%	0.130	26%	47%	0.093	38%	43%	0.643
Malperfusion, %	58%	13%	0.003	37%	18%	0.119	31%	22%	0.523
Operation data									
Bentall procedure	7	25	< 0.001	3	28	0.350	1	31	0.175
David procedure	5	1		1	4		1	5	
CABG	2	0	0.186	0	1	0.817	1	1	0.237
Ascending-femoral bypass	2	2	0.626	1	3	0.559	3	4	0.042
Surgery time, h	6.81±1.52	6.63±1.39	0.525	7.67±1.26	6.87±1.35	0.004	7.61±1.20	6.68±1.48	0.017
CPB time, min	175.63±41.74	150.44±31.91	0.001	171.36±35.20	156.43±38.20	0.179	172.64±31.17	158.53±38.27	0.244
Cross-clamping time, min	96.34±28.37	88.51±19.97	0.120	97.14±20.58	90.14±23.02	0.292	94.45±29.84	91.35±22.79	0.682
MHCA time, min	21.43±4.33	21.72±5.03	0.782	21.79±3.45	21.38±4.72	0.765	23.00±5.48	21.40±4.53	0.289
Blood transfusion, ml	929.33±461.29	1075.00±360.16	0.064	957.14±516.49	1043.55±382.83	0.385	946.88±514.37	1028.18±398.49	0.465
RBC, U	3.36±1.97	4.49±2.01	0.011	3.48±2.14	4.30±2.00	0.096	3.81±2.17	4.15±2.03	0.534
Plasma, ml	403.85±251.24	422.06±244.85	0.690	432.98±235.27	380.95±287.44	0.381	337.5±289.54	426.13±239.59	0.181
Platelet, U^*^	1.85±0.60	1.61±0.54	0.773	4.19±1.46	1.14±0.36	0.054	2.50±1.12	1.58±0.41	0.429
Cryoprecipitate, U^*^	1.48±0.53	2.02±0.55	0.483	1.48±0.70	1.77±0.45	0.776	2.81±0.99	1.72±0.40	0.334
Laboratory parameters at admission									
Hb, g/L	128.78±24.59	133.17±24.79	0.367	124.35±20.50	131.44±24.82	0.273	128.54±24.97	129.93±24.66	0.849
WBC, 10^9/L	11.27±4.21	12.61±5.75	0.173	12.47±5.69	11.34±4.08	0.333	13.13±8.97	11.51±4.21	0.271
PLT, 10^9/L	167.22±53.43	156.59±52.83	0.312	144.65±67.80	164.21±49.26	0.166	148.31±66.16	161.77±51.91	0.397
AST, U/L	20.00 [18.00,26.00]	24.50 [19.25,43.75]	0.027	21.00 [18.00,26.00]	27.00 [30.00,43.50]	0.670	25.00 [17.50,61.75]	22.00 [18.50,26.50]	0.371
ALT, U/L	24.50 [19.25,43.75]	34.50 [27.00,42.75]	0.050	30.00 [25.00,38.00]	32.00 [27.50,43.50]	0.655	28.50 [20.25,49.50]	31.00 [51.50,111.50]	0.370
TBIL, µmol/L	19.35±8.01	21.61±13.62	0.294	19.65±9.18	20.45±10.86	0.776	17.18±10.08	20.53±10.56	0.299
DBIL, µmol/L	5.79±2.25	7.06±3.99	0.062	7.47±3.82	6.07±2.95	0.094	6.49±2.60	6.39±3.19	0.913
IDBIL, µmol/L	13.39±8.26	14.54±12.25	0.569	12.18±7.93	14.26±10.44	0.439	10.68±8.20	14.04±10.11	0.271
BUN, mmol/L	8.02±4.74	8.69±4.01	0.450	10.12±5.23	7.92±4.20	0.063	9.11±3.31	8.47±4.70	0.652
Cre, µmol/L	66.00 [48.00,105.00]	82.50 [61.00,107.25]	0.274	68.50 [47.50,96.00]	86.00 [64.50,126.00]	0.181	101.00 [82.5,181.00]	72.00 [51.50,111.50]	0.716
FIB, g/L	2.60 [2.02,3.92]	2.30 [1.79,3.26]	0.060	2.52 [2.00,3.84]	1.99 [1.67,2.94]	0.238	2.02 [1.62,3.09]	2.48 [1.99,3.75]	0.099
DD, mg/L	7.16 [1.87,11.02]	12.97 [6.83,30.78]	0.011	7.70 [2.12,17.10]	10.90 [7.78,18.86]	0.654	34.75 [5.24,53.61]	8.05 [2.48,17.10]	0.026
FDP, mg/L	21.72 [5.75,33.13]	39.40 [21.62,87.76]	0.005	24.25 [7.07,52.25]	37.03 [23.97,62.03]	0.736	75.32 [15.76,22.12]	25.18 [8.64,50.55]	0.026
CRP, mg/L	14.90 [5.95,48.10]	6.65 [3.40,60.68]	0.857	10.46 [4.96,48.60]	51.30 [9.87,108.20]	0.360	6.19 [0.91,51.3]	11.70 [5.34,53.25]	0.429
PCT, ng/mL	0.24 [0.13,0.50]	0.39 [0.10,1.60]	0.088	0.24 [0.10,0.57]	1.10 [0.35,1.33]	0.775	0.32 [0.12,0.71]	0.34 [0.10,1.00]	0.476
Postoperative laboratory parameters									
Hb, g/L	103.92±19.32	101.93±13.38	0.526	101.48±17.00	102.53±15.63	0.784	101.36±22.23	102.77±15.30	0.758
WBC, 10^9/L	12.40±3.79	12.79±4.28	0.322	13.54±4.44	12.39±3.98	0.127	12.36±3.20	12.68±4.08	0.356
PLT, 10^9/L	69.60±36.17	75.96±33.55	0.607	62.81±38.40	75.49±33.20	0.246	64.21±32.07	73.28±34.80	0.783
AST, U/L	76.50 [45.50,185.50]	53.50 [40.25,79.25]	0.008	86.00 [50.50,247.00]	57.00 [40.75,83.25]	0.598	97.00 [62.75,440.75]	59.00 [41.00,97.00]	0.225
ALT, U/L	37.50 [27.00,76.50]	31.00 [26.00,44.00]	0.021	37.00 [28.50,70.50]	32.00 [26.00,44.25]	0.766	55.50 [28.75,134.00]	32.00 [26.00,48.00]	0.323
TBIL, µmol/L	49.98±23.53	42.70±24.11	0.100	55.82±28.85	43.85±22.62	0.040	43.05±19.84	45.73±24.22	0.692
DBIL, µmol/L	20.21±11.91	15.78±13.01	0.057	23.10±18.15	16.57±11.20	0.036	16.89±8.04	17.54±12.99	0.856
IDBIL, µmol/L	29.77±16.84	26.92±14.25	0.318	32.72±17.54	27.27±14.63	0.140	26.16±18.42	28.20±14.93	0.640
BUN, mmol/L	14.92±5.35	12.96±5.00	0.041	16.33±5.89	13.30±4.94	0.016	14.25±5.34	13.73±5.19	0.727
Cre, µmol/L	166.50 [104.50,244.75]	104.50 [80.00,163.00]	0.094	162.00 [103.50,211.50]	109.50 [80.00,191.75]	0.281	176.50 [108.25,253.25]	115.00 [86.00,194.00]	0.426
FIB, g/L	2.72 [10.92,3.17]	3.08 [2.55,3.64]	0.001	2.27 [1.81,2.91]	3.04 [2.58,3.58]	0.001	1.99 [1.79,3.00]	2.99 [2.48,3.48]	0.003
DD, mg/L	15.86 [10.92,20.05]	16.19 [8.86,20.36]	0.277	16.14 [11.57,21.85]	15.84 [9.00,20.10]	0.576	15.88 [14.14,26.76]	15.90 [9.55,20.11]	0.070
FDP, mg/L	59.23 [42.61,77.37]	54.78 [36.28,77.43]	0.369	57.73 [38.16,80.35]	55.41 [39.96,75.00]	0.951	59.23 [45.90,99.77]	55.04 [37.28,77.16]	0.248
CRP, mg/L	97.40 [75.23,145.10]	114.60 [49.50,160.40]	0.667	103.95 [61.48,135.53]	105.60 [67.50,156.40]	0.424	113.05 [70.55,155.43]	82.35 [27.28,119.13]	0.047
PCT, ng/mL	9.70 [4.15,17.00]	7.70 [2.80,16.00]	0.572	11.50 [3.97,21.25]	9.00 [3.40,16.00]	0.846	7.50 [2.40,21.00]	9.40 [3.40,16.00]	0.714
IL-6	134.57±73.24	104.60±80.07	0.291	119.69±77.72	113.52±80.84	0.866	145.53±17.99	113.08±78.44	0.568

ALT=alanine aminotransferase; AST=aspartate aminotransferase; BMI=body mass index; BUN=blood urea nitrogen; CABG=coronary artery bypass grafting; CKD=chronic kidney disease; CPB=cardiopulmonary bypass; Cre=creatinine; CRP=C-reactive protein; DBIL=direct bilirubin; DD=D-dimer; DM=diabetes mellitus; FDP=fibrinogen degradation products; FIB=fibrinogen; Hb=hemoglobin; HTN=hypertension; IDBIL=indirect bilirubin; IL-6=interleukin-6; MHCA=mild hypothermic circulatory arrest; PCT=procalcitonin; PLT=platelet; RBC=red blood cell; TBIL=total bilirubin; WBC=white blood cell

^*^Platelet and cryoprecipitate were expressed as mean + standard error

**Table 2:** Perioperative immune-inflammatory parameters by different clinical outcomes.

Index^*^	Delayed extubation			Reintubation			30-day mortality		
	Yes	No	*P*-value	Yes	No	*P*-value	Yes	No	*P*-value
Pre. SIRI	8.36 [3.70,15.46]	5.84 [3.38,7.82]	0.025	6.02 [3.41.10.10]	7.04 [4.56,15.30]	0.743	10.92 [3.57,19.61]	6.28 [3.58,9.79]	0.297
Pre. SII	2219.9 [1321.4,3165.5]	1344.69 [989.44,2449.30]	0.063	1720.5 [1072.3,3343.5]	1892.6 [1039.7,2595.5]	0.890	2612.6 [719.8,3908.3]	1874.1 [1039.7,2599.3]	0.577
Pre. ALI	6.18 [3.79, 12.21]	9.22 [5.77,14.00]	0.153	9.06 [4.71,12.23]	8.20 [5.12,13.09]	0.268	6.60 [3.48,8.74]	8.39 [5.20,12.38]	0.166
Pre. PNI	38.94±5.84	41.78±4.39	0.006	38.87±5.69	40.96±4.86	0.105	38.49±7.14	40.50±5.08	0.220
Pre. PIV	1171.9 [668.8,2356.4]	801.61 [510.74,1208.55]	0.039	940.5 [563.3,1699.2]	939.6 [688.8,2463.4]	0.533	1659.8 [265.0,3298.5]	1427.3 [960.2,2680.3]	0.250
Post. SIRI	12.72 [9.33,15.16]	12.52 [9.87,16.61]	0.295	12.52 [9.43,15.80]	12.11 [9.73,17.56]	0.769	11.89 [8.86,14.33]	12.60 [9.85,15.93]	0.547
Post. SII	1466.1 [668.8,2356.4]	1428.5 [1150.0,2845.8]	0.678	1165.7 [548.4,2178.3]	1437.7 [1083.0,2780.4]	0.079	1065.6 [388.6,1820.2]	1427.3 [960.2,2680.3]	0.099
Post. ALI	4.07 [2.91,7.86]	4.29 [3.08,6.07]	0.966	4.60 [3.08,7.42]	4.10 [2.99,6.20]	0.782	4.89 [3.41,8.76]	4.13 [3.04,6.18]	0.417
Post. PNI	40.05±4.89	42.55±4.13	0.003	39.51±5.95	42.01±4.30	0.027	36.31±6.82	41.93±4.18	0.009
Post. PIV	757.1 [351.0,1497.7]	934.0 [553.8,1877.6]	0.480	803.3 [192.1,1538.5]	889.5 [548.1,1814.5]	0.341	565.1 [130.8,1501.6]	910.2 [541.6,1726.6]	0.464

ALI=advanced lung cancer inflammation index; PIV=pan-immune-inflammation value; PNI=prognostic nutritional index; SII=systemic immune-inflammation index; SIRI=systemic inflammation response index

^*^Pre. Stands for preoperative values and Post. Represents the parameters within 24 hours after surgery

### Risk Factors for Poor Clinical Outcomes

By multivariate logistic regression analysis adjusted for age, sex, BMI, history of diseases, smoking, drinking, and preoperative malperfusion, postPNI and postFIB were the two protective parameters of poor clinical outcomes. The odds ratios (ORs) (95% confidence interval [CI]) of postPNI were 0.898 (0.815, 0.989) for delayed extubation and 0.792 (0.696, 0.901) for 30-day mortality. The ORs (95% CI) of postFIB were 0.487 (0.291, 0.813) for delayed extubation, 0.292 (0.124, 0.687) for reintubation, and 0.249 (0.093, 0.669) for 30-day mortality. CPB time was the only risk factor of delayed extubation in the multivariate logistic regression analysis (Table 3). Other immune-inflammatory parameters did not reach statistical significance even during univariate regression analysis.

**Table 3:** Prognostic parameters screened by univariate and multivariate logistic regression analysis.

Index^*^	Delayed extubation			Reintubation			30-day mortality		
	*P*-value	OR	95% CI	*P*-value	OR	95% CI	*P*-value	OR	95% CI
Univariate logistic regression									
BMI	0.047	1.122	1.002, 1.258						
Malperfusion	0.004	3.774	1.534, 9.286						
CPB time	0.006	1.017	1.005, 1.029	0.046	1.676	1.010,2.783			
Pre. DD	0.012	1.037	1.008, 1.067				0.003	1.041	1.014,1.068
Pre. FDP	0.009	1.014	1.004, 1.024				0.003	1.012	1.004,1.019
Pre. PNI	0.012	0.899	0.828, 0.977						
Post. FIB	0.001	0.423	0.253,0.705	0.008	0.292	0.118, 0.720	0.004	0.256	0.102,0.643
Post. PNI	0.016	0.903	0.832,0.981	0.040	0.902	0.817,0.995	0.001	0.811	0.720,0.913
Multivariate logistic regression^**^									
CPB time	0.020	1.016	1.003,1.030						
Post. FIB	0.006	0.487	0.291,0.813	0.005	0.292	0.124, 0.687	0.006	0.249	0.093,0.669
Post. PNI	0.029	0.898	0.815,0.989	0.072	0.908	0.817,1.009	<0.001	0.792	0.696,0.901

BMI=body mass index; CI=confidence interval; CPB=cardiopulmonary bypass; DD=D-dimer; FDP=fibrinogen degradation products; FIB=fibrinogen; OR=odds ratio; PNI=prognostic nutritional index

^*^Pre. stands for preoperative values and Post. stands for postoperative values

^**^Age, gender, BMI, history of diseases, smoking, drinking, and preoperative mulperfusion were adjusted during multivariate analysis

### Discriminating Performances of PostPNI and PostFIB in Predicting Poor Clinical Outcomes

**Fig. 2:**
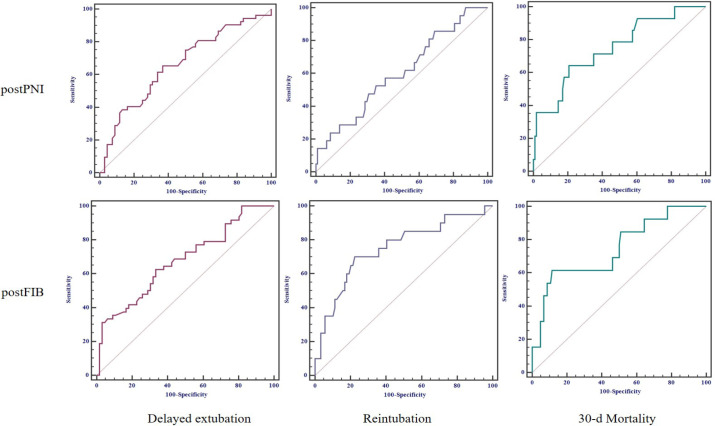
Receiver operating characteristic curves by different clinical outcomes. postFIB=postoperative fibrinogen; postPNI=postoperative prognostic nutritional index.

To determine the prognostic predictive abilities of postPNI and postFIB for a poor clinical prognosis of ATAAD after surgery, we conducted ROC analysis. The areas under the curve (AUCs) for postPNI were 0.659 (0.567, 0.743) for delayed extubation, 0.603 (0.507, 0.693) for reintubation, and 0.746 (0.661, 0.820) for 30-day mortality, and the cutoff values were 42.1, 40.3, and 38.55, respectively. The AUCs for postFIB were 0.678 (0.584, 0.762) for delayed extubation, 0.751 (0.659, 0.828) for reintubation, and 0.745 (0.656, 0.821) for 30-day mortality, and the cutoff values were 2.87, 2.54, and 2.08, respectively (Figure 2, Table 4). The predicted values of the two parameters for different clinical outcomes did not show significant differences. The combination of two parameters did not further enhance the predictive values.

**Table 4:** ROC analysis of postPNI and postFIB by different clinical outcomes.

Index	Delayed extubation		Reintubation		30-day mortality	
	postPNI	postFIB	postPNI	postFIB	postPNI	postFIB
AUC	0.659	0.678	0.603	0.751	0.746	0.745
95% CI	0.567,0.743	0.584,0.762	0.507,0.693	0.659,0.828	0.661,0.820	0.656,0.821
Sensitivity	65.4	62.5	52.4	70.0	64.3	61.5
Specificity	63.2	66.7	64.9	77.5	79.3	88.5
Cutoff value	42.1	2.87	40.3	2.54	38.55	2.08

AUC=area under the curve; CI=confidence interval; postFIB=postoperative fibrinogen; postPNI=postoperative prognostic nutritional index; ROC=receiver operating characteristic

## DISCUSSION

This study explored the prognostic predictive and discriminative abilities of different immune-inflammatory parameters, including SIRI, SII, PNI, ALI, and PIV, in ATAAD patients after surgery. The prognostic indices included delayed extubation, reintubation, and 30-day mortality. The rates of delayed extubation, reintubation, and 30-day mortality were 43.7%, 16.8%, and 13.6%, respectively. The 30-day mortality was similar to those in previous multicenter studies, which uniformly approximately 17%. We found that only low postPNI was intimately associated with delayed extubation and 30-day mortality. Other perioperative immune-inflammatory indices did not present any predictive value of poor clinical outcomes after ATAAD surgery. In addition, low postFIB could well predict poor clinical outcomes.

Aberrant activation of the immune-inflammatory system plays a pivotal role in the progression of AD, contributing to vascular remodeling and dissection formation^[14]^. In ATAAD patients, neutrophils usually secrete cytokines in response to inflammatory stimuli, and cellular immunity is weakened, which is indicated by a decrease in lymphocytes. Therefore, NLR and NLR-derived parameters could reflect the general immune-inflammatory status. In this study, preNLR and postNLR were 14.93±14.57 and 27.06±19.13, respectively, indicating the activation of inflammation. Studies have reported that NLR can distinguish AD from other acute chest pain diseases, and patients with a higher NLR tend to have higher in-hospital mortality^[8,15]^. There are few data on the relationship of SIRI, SII, ALI, and PIV with the prognosis of ATAAD after surgery. In this study, we did not find any significant differences between different groups divided by delayed extubation, reintubation, or 30-day mortality.

Previous studies have proposed albumin as an indicator of protein status in non-inflamed patients, but it is not nutritionally informative in an ICU setting. The distribution between the intravascular and extravascular compartments, the rates of synthesis, and the metabolism of albumin are all significantly altered during inflammation and stress. It was reported that the normal transcapillary escape rate for albumin increases by 100% after cardiac surgery. In addition, the transcription rate of albumin messenger ribonucleic acid is decreased in response to inflammation^[16,18]^. Anti-inflammation and immune regulation are also two important physiological roles of albumin^[18]^. Therefore, hypoalbuminemia could reflect a systemic immune-inflammatory state and further enhance the inflammatory response. A lower albumin level has predicted higher in-hospital mortality in both ATAAD and type B AD^[19]^. PNI is an effective index that integrates two inflammatory markers - serum albumin and lymphocytes. Previous studies reported that PNI was independently associated with all-cause and cardiovascular mortality in patients hospitalized for acute heart failure, coronary artery disease, or infective endocarditis^[21,22]^. Similar prognostic predictive values have been observed for PNI in patients after cardiac surgery, including CABG or aortic valve replacement^[22,24]^. Recently, several studies revealed its intimate association with ATAAD. Low PNI at admission has been strongly correlated with in-hospital mortality in patients after surgery, especially in hypertensive patients, even after adjusting for other risk factors^[10,11]^. Though we found that prePNI was lower in patients with delayed extubation, it was not an independent risk factor after multivariate analysis. This discrepancy might be attributed to the different populations, statistical methods, and surgical processes. Furthermore, those studies did not assess the influence of postPNI on prognosis. In this study, we found that low postPNI well predicted poor clinical outcomes after multivariate logistic regression analysis. PostPNI was significantly lower in the groups with the poor clinical outcomes of delayed extubation or 30-day mortality.

Inflammation is an important regulator of coagulation and fibrinolytic system activity. Acute inflammation is known to shift the hemostatic balance toward a prothrombotic and antifibrinolytic state, and FIB could also be a driver of local inflammation^[25]^. An animal study showed that FIB was oxidized at first and proteolyzed three hours later in response to leukocyte-associated inflammation^[26]^. Changes in coagulation and fibrinolysis are prominent in ATAAD patients due to acute inflammatory response, endothelial injury, formation of false lumen, and thrombosis. A Swedish study described that FIB levels at admission were significantly lower in ATAAD patients than in patients undergoing surgery of the ascending aorta or the aortic root in mild-tomoderate hypothermia^[27]^. The levels of FIB further decreased after CPB. Low FIB (< 2.17 g/L) at admission was reported to be an independent predictor of in-hospital mortality in patients undergoing ATAAD surgery, especially in patients > 65 years^[28]^. However, few studies have discussed the influence of postFIB. We found that low postFIB was strongly associated with delayed extubation, reintubation, and 30-day mortality after adjusting for confounders in this study. These results indicate that low postFIB could well predict poor clinical outcomes and might be a promising prognostic marker of ATAAD after surgery.

### Limitations

Several limitations of this study should be stressed. It was a small, single-center retrospective study. There were few events, and local surgical skills might have influenced the clinical outcomes. Therefore, larger, multicenter, and prospective studies are required to verify our results.

## CONCLUSION

Prognostic estimation is crucial for the management of ATAAD. We found that low postPNI, rather than other perioperative immune-inflammatory indices, was intimately associated with delayed extubation and 30-day mortality. Low postFIB was strongly associated with delayed extubation, reintubation, and 30-day mortality after adjusting for confounders in this study. Overall, postPNI and postFIB might be two easily accessible and effective prognostic markers to guide the risk stratification and treatment of ATAAD patients.

**Table:** 

Authors’ Roles & Responsibilities	
JWH	Substantial contributions to the conception or design of the work; or the acquisition, analysis, or interpretation of data for the work; drafting the work or revising it critically for important intellectual content; final approval of the version to be published
TS	Substantial contributions to the conception or design of the work; or the acquisition, analysis, or interpretation of data for the work; agreement to be accountable for all aspects of the work in ensuring that questions related to the accuracy or integrity of any part of the work are appropriately investigated and resolved; final approval of the version to be published
